# Correction: Progression in training volume and perceived psychological and physiological training distress in Norwegian student athletes: A cross-sectional study

**DOI:** 10.1371/journal.pone.0266313

**Published:** 2022-03-28

**Authors:** The PLOS ONE Staff

There are errors in the Author Contributions. The correct contributions are:

**Conceptualization:** Cathrine Nyhus Hagum, Espen Tønnessen, Shaher A. I. Shalfawi

**Data curation:** Cathrine Nyhus Hagum

**Formal analysis:** Cathrine Nyhus Hagum

**Funding acquisition:** Shaher A. I. Shalfawi

**Investigation:** Cathrine Nyhus Hagum, Espen Tønnessen, Shaher A. I. Shalfawi

**Methodology:** Cathrine Nyhus Hagum, Espen Tønnessen, Shaher A. I. Shalfawi

**Project administration:** Shaher A. I. Shalfawi

**Supervision:** Espen Tønnessen, Shaher A. I. Shalfawi

**Validation:** Cathrine Nyhus Hagum, Espen Tønnessen, Shaher A. I. Shalfawi

**Visualization:** Cathrine Nyhus Hagum, Espen Tønnessen, Shaher A. I. Shalfawi

**Writing–original draft:** Cathrine Nyhus Hagum

**Writing–review & editing:** Cathrine Nyhus Hagum, Espen Tønnessen, Shaher A. I. Shalfawi

The publisher apologizes for the errors.
