# Supplementary material for: Progression in training volume and perceived psychological and physiological training distress in Norwegian student athletes: A cross-sectional study
Source: PLoS One. 2022 Feb 4;17(2):e0263575. doi: 10.1371/journal.pone.0263575 (PMC8815906; doi:10.1371/journal.pone.0263575)
Supplement: S1 Table — (DOCX) [file pone.0263575.s001.docx]

**S1 Table**. **Pearson bivariate correlations among study variables.** Male are above the diagonal (n = 308) and female are below the diagonal (n = 298).

|  | DEP | VIG | SYM | SLE | STR | FAT |
| --- | --- | --- | --- | --- | --- | --- |
| DEP | - | 0.269** | 0.083 | 0.275** | 0.436** | 0.381** |
| VIG | 0.204** | - | -0.002 | 0.147** | 0.187** | 0.211** |
| SYM | 0.346** | 0.030 | - | 0.208** | 0.163** | 0.343** |
| SLE | 0.424** | 0.233** | 0.256** | - | 0.273** | 0.380** |
| STR | 0.638** | 0.218** | 0.269** | 0.392** | - | 0.372** |
| FAT | 0.524** | 0.214** | 0.351** | 0.467** | 0.539** | - |

* *p* <.05, ** *p* <.01
