# Supplementary material for: Progression in training volume and perceived psychological and physiological training distress in Norwegian student athletes: A cross-sectional study
Source: PLoS One. 2022 Feb 4;17(2):e0263575. doi: 10.1371/journal.pone.0263575 (PMC8815906; doi:10.1371/journal.pone.0263575)
Supplement: S2 Table — (DOCX) [file pone.0263575.s002.docx]

**S2 Table. The categorization of the different sports in the present study***.* In the research literature, training load is juxtaposed with the product of duration, intensity, and frequency. The categorization in the table below is made on the basis that the load in the various sports is approximately equal, as well, as they have similar sport demands. Three experts have categorized the sports equally.

| Type of Sport | Sport^a^ | *n* | % |
| --- | --- | --- | --- |
| Soccer | Soccer | 290 | 45.9 |
|  | Total | 290 | 45.9 |
| Other team- and ball sports | Handball | 90 | 14.2 |
|  | Ice hockey | 19 | 3.0 |
|  | Badminton | 5 | 0.8 |
|  | Tennis | 4 | 0.6 |
|  | Floorball | 1 | 0.2 |
|  | Volleyball | 5 | 0.8 |
|  | Total | 124 | 19.6 |
| Endurance sports | Swimming | 24 | 3.8 |
|  | Cross-country skiing | 34 | 5.4 |
|  | Orienteering | 8 | 1.3 |
|  | Cycling | 12 | 1.9 |
|  | Rowing | 3 | 0.5 |
|  | Biathlon | 11 | 1.7 |
|  | Triathlon | 2 | 0.3 |
|  | Total | 94 | 14.9 |
| Weight-bearing sports | Track & Field/ Athletics | 21 | 3.3 |
|  | Gymnastics | 11 | 1.7 |
|  | Alpine skiing | 15 | 2.4 |
|  | Strength training | 4 | 0.6 |
|  | Freeski | 1 | 0.2 |
|  | Total | 52 | 8.2 |
| Other sports | Golf | 3 | 0.5 |
|  | Show Jumping | 12 | 1.9 |
|  | Ice Skate | 4 | 0.6 |
|  | Sailing | 6 | 0.9 |
|  | Martial Art | 7 | 1.1 |
|  | Cheerleading | 1 | 0.2 |
|  | Sky Jumping | 1 | 0.2 |
|  | Diving | 1 | 0.2 |
|  | Sports drill | 4 | 0.6 |
|  | Shooting | 1 | 0.2 |
|  | Snowboard | 1 | 0.2 |
|  | Jetski | 1 | 0.2 |
|  | Dance | 1 | 0.2 |
|  | Motocross | 2 | 0.3 |
|  | Climbing | 1 | 0.2 |
|  | Figure skating | 1 | 0.2 |
|  | Total | 47 | 7.7 |

*n* = sample size
^a^1 missing value was observed for sport
