# Supplementary material for: Progression in training volume and perceived psychological and physiological training distress in Norwegian student athletes: A cross-sectional study
Source: PLoS One. 2022 Feb 4;17(2):e0263575. doi: 10.1371/journal.pone.0263575 (PMC8815906; doi:10.1371/journal.pone.0263575)
Supplement: S1 File — (DOCX) [file pone.0263575.s003.docx]

**S1 File. Testing normality.** Information regarding the normality assumption for running MANOVA.

The normality assumption was examined by conducting Shapiro-Wilk (SW) and Kolmogorov-Smirnov (KS) tests. A statistically significant (*p*<0.05) test would indicate not normally distributed data (Verma, 2015, p. 57). A limitation of these tests is that they can become significant even for a slight deviation from normality in the case of large samples (Verma, 2015, p. 55). Hence, normal Q-Q plots were also investigated to check the level of normality. The SW and KS tests for both males and females were statistically significant (*p*<.001) for all dependent variables, indicating nonnormal data distribution. However, the points in the Normal Q-Q plot were along the line for vigor, physical symptoms, stress, and fatigue, for both males and females, indicating a normal data distribution. The points had minor deviations from the line for depression and sleep disturbances for both males and females. The box-plot indicated some outliers, but these were not considered as outliers because the range of responses was small (i.e., one to five). Similarly, for the training hours classifications, the SW and KS tests were statistically significant (*p*<.001). However, when inspecting the QQ-plots, the points had only small deviations from the line. No outliers were found in the training load classification data. It was not surprising that the SW and KS tests were statistically significant, given the relatively large sample size in the present study. Considering that the MANOVA analysis is robust against the violation of normality (Verma, 2015, p. 210), we determined that it would be safe to proceed with further analysis.
