# Supplementary material for: Progression in training volume and perceived psychological and physiological training distress in Norwegian student athletes: A cross-sectional study
Source: PLoS One. 2022 Feb 4;17(2):e0263575. doi: 10.1371/journal.pone.0263575 (PMC8815906; doi:10.1371/journal.pone.0263575)
Supplement: S2 File — (DOCX) [file pone.0263575.s004.docx]

**S2 Information.** **Descriptive discriminant analysis.**

The DDA evaluating the effect of gender on training distress showed that the Box’s M test was not statistically significant (*p*>.001). Further, the log determinants were relatively similar, so the assumption of homogeneity of variance-covariance was considered to be met.

To save the discriminant function scores for an interaction effect, we followed the guidelines proposed in DDA literature (Barton et al., 2016; Enders, 2003; Smith et al., 2019). The Box’s M test was not statistically significant (*p*>.001), and the log determinants ranged between -5.22 and -3.01. The log determinant of the pooled covariance matrix was -3.51. As such, the assumption of homogeneity of variance/ covariance was considered to be met.

A two-way ANOVA was run to determine which groups were different on the interaction composite. We created a multivariate interaction composite, which was used as the dependent variable. Simple comparisons were conducted to examine differences among training groups within each school level. A Bonferroni adjustment was used to control for Type 1 error inflation across the set of three comparisons (i.e., *α* = .05/3 = .016).
