# Supplementary material for: Progression in training volume and perceived psychological and physiological training distress in Norwegian student athletes: A cross-sectional study
Source: PLoS One. 2022 Feb 4;17(2):e0263575. doi: 10.1371/journal.pone.0263575 (PMC8815906; doi:10.1371/journal.pone.0263575)
Supplement: Attachment — Submitted filename: Response to Reviewers.docx [file pone.0263575.s006.docx]

**Response to Reviewers**

Dear Dr. Jonathan Smith,
Thank you for the opportunity to submit a revised version of the manuscript titled Progression in training volume and perceived psychological and physiological training distress in Norwegian student athletes to *PLOS ONE*. We appreciate the time and effort you and the reviewers have dedicated to providing valuable feedback on the submitted manuscript. We have addressed the raised concerns and hope our responses and revisions will meet your expectations. We have highlighted the changes within the manuscript.

Below is a point-by-point response to the academic editor and the reviewers' comments and concerns. All changes in the manuscript are marked up with red colour.

**Journal requirements**

1. Please ensure that your manuscript meets PLOS ONE's style requirements, including those for file naming. The PLOS ONE style templates can be found at <https://journals.plos.org/plosone/s/file?id=wjVg/PLOSOne_formatting_sample_main_body.pdf> and <https://journals.plos.org/plosone/s/file?id=ba62/PLOSOne_formatting_sample_title_authors_affiliations.pdf>.

**Response to the academic editor:**
We have double-checked the manuscript to meet PLOS ONE's style requirements.

2. We noted in your submission details that a portion of your manuscript may have been presented or published elsewhere. (Hagum and Shalfawi, 2020) Please clarify whether this [conference proceeding or publication] was peer-reviewed and formally published. If this work was previously peer-reviewed and published, in the cover letter please provide the reason that this work does not constitute dual publication and should be included in the current manuscript.

**Response to the academic editor:**
In the submitted research article, we have used the same data as in Hagum and Shalfawi (2020), which is peer-reviewed and published. However, Hagum and Shalfawi (2020) is a methodological article investigating the factorial validity of the MTDS-N questionnaire. The current research article raises a new set of research questions which was part of the pre-approved protocol by the Norwegian Social Science Data Services (NSD) (Project number: 836079) and the Regional Committees for Medical and Health Research Ethics (REK) (project number: 54584) prior to data collection. Hence, this work does not constitute dual publication.

**Reviewer #1**

**Review Comments to the Author**
This manuscript was clear, concise, and easy to read, and the methods were appropriate for the purposes of the study. Furthermore, the authors provided practical implications for coaches and sport administrators based on their results and other literature. I have a few suggestions to further strengthen this paper.

**Response to the reviewer:**
Thank you for taking the time to assess the manuscript. We have addressed the raised concerns and hope our responses and revisions will meet your expectations.

Methods:

1. Please include a little more detail on the procedure so that readers don't have to look up the other paper. At a minimum, I'd like to know at what point in the school year data collection took place, and where/how the questionnaires were completed (e.g., during class, at sport practice, online, etc.)

   **Response to the reviewer:**
   We thank the reviewer for pointing this out. We have updated the manuscript with the following text: All upper secondary schools that offer the optional program subject top-level sport in Norway (*n* = 119) were invited to participate in the present study. The MTDS-N was distributed electronically using SurveyXact version 8.0 (Ramboll, n.d.) to the school management who agreed to participate (*n* = 34, 28.6%). Further, the school management distributed the questionnaire electronically to the student-athletes at their respective schools (*n* = 23, 19.3%). The data collection took place during class and started in March 2020 and ended in May 2020. To assess training volume, student athletes reported their current weekly training hours. In addition, the survey included questions regarding age, gender, county, school name, study program, school level, and main type of sport.
2. The categories of soccer, other team- and ball sports, and endurance sports make sense, but I'm not convinced by the separation of sports into "weight-bearing" and "other." Surely gymnastics and cheerleading (and perhaps figure skating and diving) share more similarities than gymnastics and track and field? And shouldn't snowboarding go in the same category as alpine skiing?

**Response to the reviewer:**
This is an important and reasonable argument. It was challenging to place some of the sports in the present study. The different sports included in the "weight-bearing" category had the most common features concerning the type of load dynamics, muscle contraction, and duration. Performance in these sports requires maximum power of both the anaerobic alactic system and the anaerobic lactic system. These were the criteria that we considered when placing them in this category. We could have done it in other ways, but this was the choice we made.

We considered placing snowboard together with alpine skiing. However, the choice fell on "other" because of the number of participants reporting snowboarding as their main sport and since snowboarding involves more acrobatic elements than requirements for strength and load dynamics as in the other sports placed in the "weight-bearing" category.

A reason for creating a category with "other" sports was that several sports had a low *n*. For example, all sports in the category "other" have an *n* ranging between 0.2-1.9% of the total sample size of 608 participants. To do sensible statistical analyses, we had to ensure enough participants in each sport category.

S2 Table, "The categorization of the different sports in the present study," is updated with track changes. In this table, we had forgotten to implement five different sports (jetski, dance, motocross, climbing, and figure skating) in the "other sports" category.

Results:

1. Please give effect sizes (e.g., partial eta squared) for the ANOVAs and MANOVAs. **Response to the reviewer:**
   We agree and have presented effect sizes by calculating Cohen's *d* for the ANOVAs (please see lines 212 to 218) and the two-way ANOVA (please see lines 239 to 245). Further, effect sizes for the MANOVAs are presented with squared canonical correlation (*R*^2^_c_), which is inverse of Wilks' lambda. This variance-accounted-for effect size is analogous to R^2^ in multiple regression or eta squared (η^2^) in ANOVA (Sherry, 2006).
2. It would be nice to have meaningful/significant mean differences indicated in the tables and figures in some way.
    **Response to the reviewer:**
   We have made sure that the meaningful/significant mean differences are explicitly stated in the manuscript text. Due to the journal requirements, the results could not be double presented. We are hoping to meet your expectations.

Discussion:

1. Line 276 – Add an apostrophe after athletes.
    **Response to the reviewer:**
   We have made the change, and an apostrophe after athletes in line 336 is added in the manuscript.
2. Lines 291-295 – The authors describe the training volume of the athletes in the weight-bearing category as unexpected, but I wonder if this is due to the inclusion of gymnastics in this category. Gymnastics is known for being an early specialization sport that requires high volumes of training from a fairly young age.
    **Response to the reviewer:**

We thank the reviewer for this valuable interpretation. We agree and have updated the manuscript: A possible explanation for this finding is that gymnastics was included in the weight-bearing category and is a sport requiring high training volume for high-level performance (Caine & Harringe, 2013).

3. Gender Differences – I've read that adult women generally have poorer sleep quality and more sleep disturbances compared to men. This might be worth mentioning, particularly if this gender difference also applies in adolescents and/or athletes.
    **Response to the reviewer:**
   This observation is correct. We have included the following text in the section discussing gender differences: Studies have also indicated that sleep disturbances are more prevalent in adolescent females (Galland et al., 2017; Hysing et al., 2013), with gender differences emerging after menses onset (Johnson et al., 2006). The female gender has also been identified as a risk factor for sleep disturbances in athletes (Walsh et al., 2021).
4. Page 22 – Although limitations are clearly stated and practical implications are provided, there isn't much in the way of suggestions for future research. Please discuss more explicitly some potential avenues for future investigations.
    **Response to the reviewer:**
   We agree and have updated the section "Limitations" with potential avenues for future research: Future research should use a longitudinal design with student athletes reporting daily training and weekly perceived training distress with the MTDS questionnaire. This could make it possible to detect spikes in perceived training distress and improve the periodization of training in student athletes. Future research should also focus on other factors explaining performance development, such as general life load and the prevalence of injury and health problems.

**Reviewer #2**

**Review Comments to the Author**
I consider that the subject addressed in this paper is interesting and relevant to improve periodization of training in young population. Although the design and the analysis developed are appropriate, several major issues about the manuscript should be addressed to improve writing quality and to fulfill STROBE (Strengthening the reporting of Observational studies in Epidemiology) guidelines for cross-sectional studies.
Various sections should be improved to guarantee a better interpretation of the information. Specific comments are presenting next.

**Response to the reviewer:**
Thank you for taking the time to assess the manuscript. We have addressed the raised concerns and hope our responses and revisions will meet your expectations.

1. To fulfill STROBE (Strengthening the reporting of Observational studies in Epidemiology) guidelines, it should be indicated the study's design with a commonly used term in the title or the abstract.
    **Response to the reviewer:**
   The title is adjusted, and the study's design is now included in the title: "Progression in training volume and perceived psychological and physiological training distress in Norwegian student athletes: a cross-sectional study." The study's design is also included in the abstract (please see line 32).
2. In addition to the objectives, it should be stated any pre-specified hypotheses in the Introduction section.

**Response to the reviewer:**
We thank the reviewer for pointing this out. We agree and have updated the manuscript. Please see lines 105 to 116.

In the section "Conclusion," we have answered the different hypotheses.

3. In Methods section the key elements of study design should be presented, including relevant dates and periods of recruitment.

**Response to the reviewer:**
We agree and have updated the manuscript. Relevant dates and periods of recruitment are included in the manuscript. Please see lines 149 to 158.

4. It should be explained the sample size calculation and how the study size was arrived at.
    **Response to the reviewer:**
   We have revised the manuscript and implemented a paragraph explaining the sample size calculation (lines 119-132): The point of stability approach was used to estimate the sample size (Hirschfeld et al., 2014; Kretzschmar & Gignac, 2019; Schönbrodt & Perugini, 2013). This approach ensures that the deviation between the estimated sample and the population parameter is stable (small) and is predicted to remain small at a stable statistical power (80%) (Kretzschmar & Gignac, 2019; Schönbrodt & Perugini, 2013). According to Cohen (Cohen, 1988), to ensure a small stability, the corridor of stability should not exceed a small correlation of 0.10. Schönbrodt and Perugini (Schönbrodt & Perugini, 2013) suggested that the minimum number needed to reach the point of stability would be 240*–*250 participants. According to Kretzschmar and Gignac (Kretzschmar & Gignac, 2019) the point-estimates of the correlation was stabilized at a sample size of 220 with perfect reliability (omega, ω = 1.0) of both latent factors and a population correlation of *p* = 0.20. Because perfect reliability is almost never attained, the authors proposed that the required sample at a population correlation of *p* = 0.20 and reliability of ω = 0.7 would be ≥ 490 participants (Kretzschmar & Gignac, 2019). Hirschfeld, Brachel and Thielsch have reported similar results with the recommended sample size to reach a point of stability was > 500 participants (Hirschfeld et al., 2014). Consequently, the sample size that was required in this study was to be more or equal to the recommendations from comparable studies (i.e., *n* ≥ 500).
5. In results section, sociodemographic characteristics of the sample should be explained.

   **Response to the reviewer:**
   We have included a table (Table 1) that explains the sociodemographic characteristics of the sample.
6. In addition to the study limitations, the strengths of the study should be indicated.
    **Response to the reviewer:**
   We have adjusted the section and implemented the strengths of the study.
7. Maybe the sections conclusions and implications for practice could be separated to properly understand the specific information of both sections.
    **Response to the reviewer:**
   We agree with the reviewer and have separated the paragraph.

Abstract

1. Lines 20 to 21: Authors only presented the instrument used to evaluate perceived training distress, but did not present the instrument used to evaluate training volume, which is also an important variable of the study. Please indicate how training volume was evaluated in the abstract.
    **Response to the reviewer:**
   We have updated the abstract (please see line 32) and the "instrument and procedure" section, explaining how we collected information about the student athletes training volume: To assess training volume, student athletes reported their current weekly training hours. In addition, the survey included questions regarding age, gender, county, school name, study program, school level, and main type of sport.
2. Line 22: (M age = 17.29, SD = .94). To maintain the style used in results section, please presented this result as M ± SD.
    **Response to the reviewer:**
   The manuscript is adjusted, and the result is presented as M ± SD.
3. Line 24: "No significant differences were found for gender, school level, or school program". I can interpret that the variable authors are analyzing is training volume, but it must be indicated in the text.

   **Response to the reviewer:**
   The manuscript is adjusted, and training volume is indicated in the text.

Introduction:

1. Authors should summarize the information to highlight the main arguments for avoiding that secondary information get reader distracted from the main argument.
    **Response to the reviewer:**
   A major part of the introduction is rewritten, maintaining the purpose of the study in mind.

Methods:

1. The design of this study should be specifically explained, including relevant dates of data collection and periods of recruitment. Maybe it would be appropriated including a subsection called "Study design" previously to "Participants" subsection.

**Response to the reviewer:**
The design of the study is presented in the title and the abstract (please see line 32). Further, we have included relevant data collection dates and recruitment periods: All upper secondary schools that offer the optional program subject top-level sport in Norway (*n* = 119) were invited to participate in the present study. The MTDS-N was distributed electronically using SurveyXact version 8.0 (Ramboll, n.d.) to the school management who agreed to participate (*n* = 34, 28.6%). Further, the school management distributed the questionnaire electronically to the student-athletes at their respective schools (*n* = 23, 19.3%). The data collection took place during class and started in March 2020 and ended in May 2020.

2. Lines 92 to 94: it should be stated the reasons why potential participants with ≤4 hours and ≥30 hours of training per week were excluded. The first criterion seems obvious for guarantee a minimum training volume, but why authors established a maximum training volume. Is this decision based in scientific evidence? If the answer is yes, please provide a reference.
    **Response to the reviewer:**
   In the preliminary analyses, SPSS marked two participants (with 40 and 37.5 hours of training a week) as outliners. One participant with 30 hours of training a week was marked as an extreme value. This participant reported soccer as the main sport. Soccer players of this age usually have 10-15 hours of training per week. This would result in self-training up to 15-20 hours a week, which we find unlikely. Hence, we considered this as a fictitious value. The decision might have been different if the reported sport was, for example, gymnastics or swimming. We decided to exclude outliers and the participant with 30 hours of training to obtain sound analyses. Weekly training volume was self-reported and is a limitation of this study. The supplementary file 1.2 testing normality indicates no outliers after excluding these three outliers. We have adjusted the manuscript, describing why we excluded the participants with ≤4 hours and ≥30 hours of training per week.
3. Lines 102 to 105: "Data regarding age, gender, country, school name, study program, school level, type of sport and weekly training volume were collected in addition to the questionnaire. The instrument and data collection procedure are fully described in (35)". The MTDS questionnaire was fully described in this reference as the authors commented, but the questions regarding type of sport and weekly training volume must be explained in order to properly interpret the data obtained for these variables.
    **Response to the reviewer:**
   We have updated the manuscript, explaining how we collected information about the student athletes type of sport and weekly training volume: To assess training volume, student athletes reported their current weekly training hours. In addition, the survey included questions regarding age, gender, county, school name, study program, school level, and main type of sport.
4. Lines 108 to 109. Please indicate that the factor vigor is a factor from the MTDS questionnaire and requires reversing it.

**Response to the reviewer:**

We have changed the sentence to: First, the factor vigor from the MTDS questionnaire, with positive scores, was reversed.

5. Lines 123 to 124: Authors should explain the reasons why they establish a cutpoint of 5, 10 and 15 hours/week for training volume groups. Is this decision based in scientific evidence? If the answer is yes, please provide a reference.
    **Response to the reviewer:**
   This is an interesting comment from the reviewer. The cutpoint of 5, 10, and 15 hours of training per week was done to ensure relatively equal group sizes (Finch & French, 2013). Further, an equal progression in training load would provide logic in the interpretation of data. The manuscript is revised, and we have included the reason for the classification of training hours. Please see lines 178 to 180.

Results:

1. Results section should start showing the main descriptive characteristics of the sample, i.e.: % of students in each type of sport, school program, school level and another descriptive characteristics with relevance to the study aim.
    **Response to the reviewer:**
   We have included a table (Table 1) showing the main characteristics of the sample in the present study.
2. Lines 161 to 163: Authors should indicate if when applied Bonferroni adjustment it was found or not significant differences in any comparisons between subgroups.

**Response to the reviewer:**
We have adjusted the manuscript and stated that Bonferroni adjustment was applied when subgroups were compared.

Discussion:

1. When starting each paragraph authors have to interpret the results obtained instead of mainly present them to avoid repeat information from Results section.
    **Response to the reviewer:**
   We have revised the discussion by removing repeated results from the result chapter and maintain the information necessary for the discussion.
2. Lines 266 to 271. Instead of repeat the purposes of the study authors should start the discussion section highlighting the main findings of the study based on the two purposes of it.

**Response to the reviewer:**
We have removed the purposes of the study and start the discussion section by highlighting the main findings of the study.

3. Line 275. A short general statement about the practical implication of this highlight could improve this first paragraph of Discussion section.
    **Response to the reviewer:**
   We agree with the reviewer and have included a short general statement about the practical implication.
4. Line 291 to 295. Could authors establish an interpretation of this difference and provide a possible explanation of it?
    **Response to the reviewer:**
   A possible explanation for this finding can be that gymnastics is included in the weight-bearing category. Gymnastics is known for being an early specialization sport, in which young children are exposed to a high sports-specific training volume (Moeskops et al., 2019).
3. Lines 299 to 304. Weight-bearing sport and other team and ball sport have a decreasing trend and soccer has a relative flat trend, while other sports have a progression in training volume. What could explain it? Perhaps the interest of coaches in likening the training load to adult or elite players could influence it? Could authors go in depth in this fact?
    **Response to the reviewer:**
   We thank the reviewer for the input and encouragement to reflect on this finding. One possible explanation can be linked to the type of competition in the given sports. For example, soccer has a more stable training load during the year than the sports in the "other" category, which is more preparatory.

In endurance and other individual sports, a more traditional periodization is often used where one has a progression in the number of hours of training per week (Myakinchenko et al., 2020; Treff et al., 2017; Tønnessen et al., 2014). We can assume that team and ball sports have not used periodization models to the same degree because the competition season is long, and the preparation period is short. Team and ball sports often have an equal training time from week to week, while other sports are not as tied to fixed training times, which also could explain the finding.

Another explanation might be that coaches in the same sport have different philosophies regarding training load. Furthermore, the time of the data collection could explain the finding from the present study.

5. Lines 314 to 315. To maintain the style used in results section, please presented this result as M ± SD.
    **Response to the reviewer:**
   We have adjusted the manuscript, and the result is presented as M ± SD.

Conclusion:

1. Authors should specify the main findings highlighted by this study. Maybe the sections implications for practice and conclusions could be separated to properly understand the specific information of both sections.

   **Response to the reviewer:**
   We agree with the reviewer and have revised the manuscript. The main finding is highlighted. Further, we have separated the paragraph.

**Reviewer #3**

**Review Comments to the Author**
A great study, nicely executed and well written. Some minor formatting issues with the final column of Table 2.

**Response to the reviewer:**
Thank you very much.

**References**

Caine, D., & Harringe, M. L. (2013). Epidemiology of injury in gymnastics. *Gymnastics handbook of sports medicine and science*, 111-124.

Cohen, J. (1988). *Statistical Power Analysis for the Behavioral Sciences*. Routledge.

Finch, H., & French, B. (2013). A Monte Carlo comparison of robust MANOVA test statistics. *Journal of Modern Applied Statistical Methods*, *12*(2), 4.

Galland, B. C. P., Gray, A. R. B. A. B., Penno, J. M., Smith, C. P., Lobb, C., & Taylor, R. W. P. (2017). Gender differences in sleep hygiene practices and sleep quality in New Zealand adolescents aged 15 to 17 years. *Sleep Health*, *3*(2), 77-83. <https://doi.org/10.1016/j.sleh.2017.02.001>

Hagum, C. N., & Shalfawi, S. A. (2020). The Factorial Validity of the Norwegian Version of the Multicomponent Training Distress Scale (MTDS-N). *International journal of environmental research and public health*, *17*(20), 7603.

Hirschfeld, G., von Brachel, R., & Thielsch, M. (2014). Selecting items for Big Five questionnaires: At what sample size do factor loadings stabilize? *Journal of Research in Personality*, *53*, 54-63.

Hysing, M., Pallesen, S., Stormark, K. M., Lundervold, A. J., & Sivertsen, B. (2013). Sleep patterns and insomnia among adolescents: a population-based study. *Journal of sleep research*, *22*(5), 549-556. <https://doi.org/https://doi.org/10.1111/jsr.12055>

Johnson, E. O., Roth, T., Schultz, L., & Breslau, N. (2006). Epidemiology of DSM-IV insomnia in adolescence: lifetime prevalence, chronicity, and an emergent gender difference. *Pediatrics*, *117*(2), e247-e256.

Kretzschmar, A., & Gignac, G. E. (2019). At what sample size do latent variable correlations stabilize? *Journal of Research in Personality*, *80*, 17-22.

Moeskops, S., Oliver, J. L., Read, P. J., Cronin, J. B., Myer, G. D., & Lloyd, R. S. (2019). The Physiological Demands of Youth Artistic Gymnastics: Applications to Strength and Conditioning. *Strength & Conditioning Journal*, *41*(1), 1-13. <https://doi.org/10.1519/ssc.0000000000000404>

Myakinchenko, E. B., Kriuchkov, A. S., Adodin, N. V., Dikunets, M. A., & Shestakov, M. P. (2020). One-year periodization of training loads of Russian and Norwegian elite cross-country skiers.

Ramboll. (n.d.). *SurveyXact*. Retrieved March 03, 2021 from <https://www.surveyxact.com/product/>

Schönbrodt, F. D., & Perugini, M. (2013). At what sample size do correlations stabilize? *Journal of Research in Personality*, *47*(5), 609-612.

Sherry, A. (2006). Discriminant Analysis in Counseling Psychology Research. *The Counseling Psychologist*, *34*(5), 661-683. <https://doi.org/10.1177/0011000006287103>

Treff, G., Winkert, K., Sareban, M., Steinacker, J. M., Becker, M., & Sperlich, B. (2017). Eleven-week preparation involving polarized intensity distribution is not superior to pyramidal distribution in national elite rowers. *Frontiers in physiology*, *8*, 515.

Tønnessen, E., Sylta, Ø., Haugen, T. A., Hem, E., Svendsen, I. S., & Seiler, S. (2014). The road to gold: training and peaking characteristics in the year prior to a gold medal endurance performance. *PLoS One*, *9*(7), e101796.

Walsh, N. P., Halson, S. L., Sargent, C., Roach, G. D., Nédélec, M., Gupta, L., Leeder, J., Fullagar, H. H., Coutts, A. J., & Edwards, B. J. (2021). Sleep and the athlete: narrative review and 2021 expert consensus recommendations. *British journal of sports medicine*, *55*(7), 356-368.
