# Supplementary material for: Progression in training volume and perceived psychological and physiological training distress in Norwegian student athletes: A cross-sectional study
Source: PLoS One. 2022 Feb 4;17(2):e0263575. doi: 10.1371/journal.pone.0263575 (PMC8815906; doi:10.1371/journal.pone.0263575)
Supplement: Attachment — Submitted filename: Response to Reviewers.docx [file pone.0263575.s008.docx]

**Response to Reviewers**

Dear Mr. Alan Ruddick,
Thank you for the opportunity to submit a revised version of the manuscript titled Progression in training volume and perceived psychological and physiological training distress in Norwegian student athletes to *PLOS ONE*. We appreciate the time and effort you and the reviewers have dedicated to providing valuable feedback on the submitted manuscript. We have addressed the raised concerns and hope our responses and revisions will meet your expectations.

Below is a point-by-point response to the academic editor and the reviewers' comments and concerns. All changes in the manuscript are marked up with red colour.

**Journal requirements**

1. Please review your reference list to ensure that it is complete and correct. If you have cited papers that have been retracted, please include the rationale for doing so in the manuscript text, or remove these references and replace them with relevant current references. Any changes to the reference list should be mentioned in the rebuttal letter that accompanies your revised manuscript. If you need to cite a retracted article, indicate the article's retracted status in the References list and also include a citation and full reference for the retraction notice.

**Response to the academic editor:**
We have double-checked the reference list to ensure that it is complete and correct. Some changes were made and are listed below.

1. Reference 5 is exchanged to the following reference:

Soligard T, Schwellnus M, Alonso J-M, Bahr R, Clarsen B, Dijkstra HP, et al. How much is too much?(Part 1) International Olympic Committee consensus statement on load in sport and risk of injury. Br J Sports Med. 2016;50(17):1030-41.

This reference is part 1 of the reference we removed (part 2).

2. Reference 1 was listed three times in the reference list. We have adjusted the reference, and it is now listed once.
3. Reference 49 has been removed due to other more relevant references used in the same sentence (Please see line 354).
4. Reference 6 was listed twice in the reference list. We have adjusted the reference, and it is now listed once.

**Reviewer #1**

**Review Comments to the Author**
Thank you for your work in revising this manuscript. I am satisfied with the responses to my previous comments. However, since PLOS ONE does not copyedit manuscripts, I gave a bit more attention to grammar and writing style and have made some suggestions in the attached document for you to consider.

**Response to the reviewer:**
Thank you for taking the time to assess the manuscript. We have considered your comments and hope our revisions will meet your expectations.

Introduction:

1. Para 1 – I recommend opening with a more general statement and simplifying the rest of the paragraph. My suggested revision is below, which you can take or leave as you see fit. I'm not convinced you need the sentence beginning with "Newly published data."

   **Response to the reviewer:**
   We thank the reviewer for pointing this out. We have updated the manuscript with the suggested revision. Please see lines 48-60.
2. Paras 2-3 – I suggest a bit of simplification and reorganization to clarify your reasoning. Regarding Line 64, (first sentence below), I think "determining" would be a better fit than "deciding" here, as "deciding" implies a choice between a set number of options. Although PLOS ONE asks for "Vancouver" referencing and does not give much direction in terms of formatting and writing style, I think it's wise to go with the APA standard of restricting the use of "e.g.," to within parentheses and spelling it out ("For example,") when outside of parentheses. If you go with my suggestions below, please double-check all of the citation numbers.

   **Response to the reviewer:**
   Thank you for the suggestions. We have revised the paragraphs (lines 61-83) in line with your suggestions. We have double-checked all of the citation numbers.
3. Lines 106, 109, 114 – Replace "where" with "with."

**Response to the reviewer:**
We have made the change and replaced "where" with "with."

4. Lines 109, 112 – Replace "have" vs. "having."

**Response to the reviewer:**
We have made the changes and replaced "have" with "having."

5. Line 114 – Could reword as "with higher weekly training volume being associated with more training distress."

**Response to the reviewer:**
Line 109 is adjusted to "with higher weekly training volume being associated with more training distress."

Materials and methods:

1. Lines 142-144 – Update formatting (M ± SD)

**Response to the reviewer:**

We have made the change in lines 137-138.

2. Lines 178-180 – Suggested revision: "Cutpoints of 5, 10, and 15 hours of training per week were chosen to ensure relatively equal group sizes"

**Response to the reviewer:**
We have revised the manuscript following your suggestion. Please see lines 178-179.

3. Line 202 – It would be of great help to students and those of us who do primarily qualitative research if you could include a reminder here of how to interpret these effect sizes (i.e., what constitutes small, medium, and large).

**Response to the reviewer:**
We thank the reviewer for the suggestion. Please see lines 203 to 207 for a description of how the effect sizes were interpreted.

4. Line 206 – "…of the sample are presented…"

**Response to the reviewer:**
We have adjusted the manuscript.

Results:

1. Description of perceived psychological and physiological training distress – Did you include the scale for this instrument somewhere? Is it from 1-5? This would be good to include here.

**Response to the reviewer:**

We agree with the reviewer and have included information about the instrument's scale. Please see lines 143-147.

2. Line 326 – I think it would be stronger to cut the first part of this sentence and begin with "No significant differences…"
   **Response to the reviewer:**

We agree and have made the change.

3. Line 340 – Remove the comma after "sports"

**Response to the reviewer:**
We have made the change.

Discussion:

1. Lines 519-527 – This paragraph seems out of place and somewhat detracts from an otherwise strong conclusion. I would recommend incorporating this content into the results section or the first paragraph of the discussion section. For example, “No significant differences in weekly training volume were observed for gender, school program, or school level, contradicting hypotheses 1b and 1c. However, in line with hypothesis 1a, results indicated a significant difference in weekly training volume between sport types, with endurance sports having a higher training volume than more technically demanding sports.”

   **Response to the reviewer:**
   Thank you for pointing this out. We have incorporated the content into the first paragraph of the discussion section (please see lines 332-340).

**Reviewer #2**

**Review Comments to the Author**
The revised version of the article adequately addresses all issues that I previously raised. Introduction and Discussion section are written more clearly now.

**Response to the reviewer:**
Thank you very much.
