# Supplementary material for: Progression in training volume and perceived psychological and physiological training distress in Norwegian student athletes: A cross-sectional study
Source: PLoS One. 2022 Feb 4;17(2):e0263575. doi: 10.1371/journal.pone.0263575 (PMC8815906; doi:10.1371/journal.pone.0263575)
Supplement: Attachment — Submitted filename: Response to Reviewers.docx [file pone.0263575.s009.docx]

**Response to Reviewers**

**Journal requirements**

1. Please review your reference list to ensure that it is complete and correct. If you have cited papers that have been retracted, please include the rationale for doing so in the manuscript text, or remove these references and replace them with relevant current references. Any changes to the reference list should be mentioned in the rebuttal letter that accompanies your revised manuscript. If you need to cite a retracted article, indicate the article's retracted status in the References list and also include a citation and full reference for the retraction notice.

**Response to the academic editor:**
We have double-checked the reference list to ensure that it is complete and correct.

**Additional Editor Comments:**

- L59 – replace high-level with high-standard

**Response:** replaced.

- L77 – remove capacity from performance capacity – unless you are referring to a physiological concept assessed as a capacity.

**Response:** correct, we are referring to the physiological performance capacity. Adjusted to: "…the necessary adjustments to optimize the physiological performance capacity…

- I believe your hypotheses would be better written as research questions as these are not in the traditional format since you are intending to falsify the null hypothesis using statistical tests.

**Response:** we agree with the feedback considering that this study is primarily exploratory. We have changed all hypotheses to research questions. Thank you.

- L128, 149, – remove the 'level' and use 'standard' throughout.

**Response:** Top-level has been changed to top-standard throughout the manuscript.

- Can you confirm how you transformed d into r and whether or not you used r squared (note: you state this as person's r).

**Response:** Cohen's d (standardized mean difference) and r^2^ (variance-accounted-for) are related and can be converted into one another. In this study the Cohen's approximate conversion formula was used, then the r multiplied to the power of 2:

$$r=\frac{d}{\sqrt{d^{2}+4}}$$

Furthermore, this conversion was conducted to measure the relationship between the variables and the "variance-accounted-for" between variables. The paragraph was rewritten as follow:

"Cohen's *d* effect sizes were converted to Person's *r* using Cohen's approximate conversion formula to measure the relationship between variables, and *r* were then multiplied to the power of 2 (i.e., *r*^2^) to be able to estimate the "variance-accounted-for" between variable (Henson, 2006). The relationships between the variables were interpreted based on the guidelines proposed by Funder and Ozer (2019), where an *r* of 0.05 indicated a very small relationship; an *r* of 0.10 indicated a small relationship; an *r* of 0.20 indicated a medium relationship; an *r* of 0.30 indicated a large relationship; and an *r* of ≥ 0.40 indicated a very large relationship."

- L213 –No need to state P < 0.001 as you state the P value in brackets. Please state the actual P value throughout rather than P < 0.001 etc.

**Response:** The P-value in L213 was removed since the P-value was stated in brackets. However, according to the PLOSONE statistical guidelines which state: "P-values. Report exact p-values for all values greater than or equal to 0.001. P-values less than 0.001 may be expressed as p < 0.001, or as exponentials in studies of genetic associations." Hence, we reported the P-values accordingly. All changes are marked with track change.

- L215 – lower volume – please check throughout and change to less volume.

**Response:** corrected throughout the manuscript.

- L216 – Please add units to your mean values throughout. Please add the SD for these means.

**Response:** We have included units, mean values and SD. Please see lines 216-227.

- Table 1 – Please consider the use of decimal age – E.g. total = 17.29 or 17 years and 105 days or 3.5 months. I think with young adults it is important to state age as clearly as possible.

**Response:** We agree and have adjusted to years ± months.

- Table 2 – M, SD and 95% CI needs units.

**Response:** hours (h) are included in Table 2.

- Line 247 – Partial eta squared first introduced. Need to state this assessment in statistical analysis.

**Response:** The assessment is stated in statistical analysis: Partial eta squared (η_p_^2^) was used to determine the effect size and were interpreted as 0.01 = small, 0.06 = medium, or 0.14 = large (Cohen, 1988, p. 368). Please see lines 166-167.

- L247 – Larger training volume rather than higher.

**Response:** corrected throughout the manuscript.

- Table 3 – see comments for table 2 – in the discussion you refer to a qualitative descriptor. Please add this into the table.

**Response:** MTDS-N score is included in Table 3. A qualitative descriptor was also added below Table 3: ^a^ The mean score of the MTDS-N, ranging between 1–5, where 1 = never/ not at all, 2 = almost never/ a little, 3 = sometimes/ moderately, 4 = fairly often/ quite a bit, and 5 = very often/extremely.

- L257 – Different is tautological – if there are dimensions they must be different.

**Response:** the sentence was removed.

- L257 to 259 – This is a description of the data in the table and is repetitive. Please remove.

**Response:** the sentence was removed.

- L263 – Results from multivariate analysis of variance – please specific what this relates to.

**Response:** the subtitle is changed to "The effect of training volume, gender, school level, sport types, and school program on the combined characteristics of training distress".

- L265 and within this section – inconsistent use of significant figures please 2 decimal places

**Response:** corrected.

- L274 – please remove as hypothesised and present the results only.

**Response:** corrected.

- L281 – inconsistent use of parentheses to illustrate variables in MANOVA, please remove.

**Response:** corrected.

- Table 4 would be considered raw data – please consider whether the presentation of this data is essential to the manuscript – there is no explicit reference to table 4 in the discussion.

**Response:** Thank you for this observation. We have now referred to Table 4 in the discussion (L417 and L491). The DDA and the results in Table 5 are based on the results shown in Table 4. Hence, we think it is essential to the manuscript.

- L299 – units required for 'group centroids' (please also see comment on figure 2).

**Response:** the manuscript is adjusted with the following text: “Female student athletes reported higher composite variable means (i.e., training distress) (0.33 ± 1.05; CI = 0.21, 0.45) than males (-0.32 ± 0.95; CI = -0.42, -0.21)”.

- Table 5 – Please consider whether or not the information contained within the table is essential to the manuscript.

**Response:** we believe the information is essential to the manuscript. We refer to the table in the discussion (L421, L433, L492). The table gives a good overview of the contribution of each outcome variable.

- Results – please pay attention to the use of the terms highest and lowest throughout

**Response:** we have read the manuscript several times and believe that the wordings were carefully checked.

- L321 – space required between equal to and greater than sign and 15

**Response:** corrected.

- L330 – start discussion with the aims of the research, follow with significant findings of this investigation. The use of hypotheses at this point is confusing, please see earlier comment regarding hypotheses.

**Response:** in line with former comments regarding hypotheses, the manuscript was adjusted accordingly in several sections of the manuscript. All changes are marked with track change.

- L338 – this is a conclusion. Please save this for the end of the manuscript after you have created a narrative for your findings.

**Response:** agreed and removed from this paragraph and moved to the conclusion.

- L351 – replace hyphen with 'to' – as in 15 to 18.

**Response:** corrected.

- Figure 1 – please rename y-axis label as Weekly Training Volume (hours), use integers of 1 hour. School level should be replaced with school year and axis labels as 'first', 'second' and 'third'. You also need to use lines that differentiate between the sports – they are all quite similar here. Consider the use of different markers. Your means also need to be accompanied by either SD's or CI's.

**Response:** the figure is adjusted. Further, we have included markers to better differentiate between sports. The means are accompanied by SD’s.

- L369 – please explore the aspect of time of data collection in relation to your data more. Might your data be compromised by this?

**Response:** we have reformulated the paragraph to: "The periods within a training macrocycle could potentially contribute to explain this finding. It is well known that different sports have different competition periods within a training macrocycle, which might have influenced the reported training volume. Hence, athletes who are in the competition season likely have less volumes with higher intensities. In comparison, athletes in the preparatory phase may have larger volumes with lower intensities where the focus is more on technical skills and the development of the general physical base".

- L379 – the average could be the mean, mode or median please qualify

**Response:** corrected. Mean ± SD.

- L382 – please include additional evidence in your assertion that athletes might be overreaching – distress alone is not sufficient evidence for this

**Response:** additional evidence was added to the text: "Conversely, a study of 17 elite Australian rowers demonstrated a decline in performance in 5 km rowing combined by altered pacing strategy, suggesting an increase in fatigue while simultaneously the total training distress scores increased significantly following four weeks of intensified training, suggesting that the athletes may have reached a short-term performance decrements accompanied by psychological and physiological symptoms including mood disturbance (Woods et al., 2017). Similar results have been found in fourteen male cyclists during a six-week training program, where increased training distress was significantly associated with increased training load (~150% of regular training load) (Woods et al., 2018)."

- L386 – Here there is little specific relationship to the findings within your study. Please provide more context relating to your study. L393 appears to relate more to speculative practical recommendations rather than a discussion of your findings.

**Response:** this section was improved by relating the results from these studies to the results of this study. E.g., "Comparing the findings from these studies (54, 55) to the findings from this study suggest that the training load was not sufficient enough for the student athletes to reach high training distress indicated by the observed "little" to "moderate" amount of training distress scores. Furthermore, it has been demonstrated that those experiencing positive training adaptations are more likely to score highly on negative dimensions included in MTDS (56), which was not observed in this study. Such results suggest that the training load must be high enough to cause stress in order to induce the desired training adaptation."

- L401 to 420 – this part of the discussion is too statistically orientated, please simply and keep to the main findings of gender differences.

**Response:** this part was rewritten, maintaining the findings of this study and reducing the statistical terms used.

- L416 – replace 'great' with 'strong'

**Response:** corrected.

- L425 – replace higher fatigue with 'greater' fatigue

**Response:** corrected.

- L433 – please be more specific about 'changes in shape'

**Response:** corrected to "human body shape".

- L436 – increased muscle mass, larger and longer bones lead to improvements in strength. Please state the subsequent improvements in aerobic capability due an increase in Hb and link central cardiovascular adaptations due to growth.

**Response:** we have adjusted the manuscript with the following text: Conversely, males typically experience physical performance improvements during adolescence. Larger and stronger bones and increased muscle mass lead to improvements in strength (Handelsman, 2017). In addition, increased circulation hemoglobin and greater muscle mass make a significant contribution to increases in peak oxygen uptake (Armstrong & Welsman, 2019).

- L470 – as previous section the first paragraph is too statistically dominant please simplify to improve readability and interpretation.

**Response:** this part was rewritten, maintaining the findings of this study and reducing the statistical terms used.

- Figure 2 – the use of centroid's or at least the term is uncommon in sport and exercise science. In your methods section or in your caption to figure 2 please provide an explanation of a centroid in lay terms and the rationale for the use of this term. I think you need to explain the practical importance of this figure in more detail to help the reader understand the message you are trying to convey. In the discussion you make reference to training distress but this is not clear from your figure.

**Response:** the word centroid has been replaced with "composite variable means" throughout the manuscript. We have explained that training distress is the composite variable. We have changed the caption in Fig 2 to: "Linear discriminant function plot showing the interaction of weekly training volume by school year and how the training volume groups separate.” In addition, we have included the following fig legend: “The figure shows the means of each training volume group on the composite outcome variable that was created from the observed variables (i.e., training distress). To facilitate the interpretation of the figure, both the *r*_s_ and the standardized coefficients from Table 5 could be examined.”

**References**

Armstrong, N., & Welsman, J. (2019). Development of peak oxygen uptake from 11–16 years determined using both treadmill and cycle ergometry. *European journal of applied physiology*, *119*(3), 801-812.

Cohen, J. (1988). *Statistical power analysis for the behavioral sciences* (2nd ed.). Laurence Erlbaum.

Funder, D. C., & Ozer, D. J. (2019). Evaluating effect size in psychological research: Sense and nonsense. *Advances in Methods Practices in Psychological Science*, *2*(2), 156-168.

Handelsman, D. J. (2017). Sex differences in athletic performance emerge coinciding with the onset of male puberty. *Clinical endocrinology*, *87*(1), 68-72.

Henson, R. K. (2006). Effect-size measures and meta-analytic thinking in counseling psychology research. *The Counseling Psychologist*, *34*(5), 601-629.

Woods, A. L., Garvican-Lewis, L. A., Lundy, B., Rice, A. J., & Thompson, K. G. (2017). New approaches to determine fatigue in elite athletes during intensified training: Resting metabolic rate and pacing profile. *PLoS One*, *12*(3), e0173807.

Woods, A. L., Rice, A. J., Garvican-Lewis, L. A., Wallett, A. M., Lundy, B., Rogers, M. A., Welvaert, M., Halson, S., McKune, A., & Thompson, K. G. J. P. o. (2018). The effects of intensified training on resting metabolic rate (RMR), body composition and performance in trained cyclists. *13*(2).
