# Supplementary material for: Progression in training volume and perceived psychological and physiological training distress in Norwegian student athletes: A cross-sectional study
Source: PLoS One. 2022 Feb 4;17(2):e0263575. doi: 10.1371/journal.pone.0263575 (PMC8815906; doi:10.1371/journal.pone.0263575)
Supplement: Attachment — Submitted filename: Reviewer Comments for PONE-D-21-13902R1.docx [file pone.0263575.s007.docx]

**Reviewer Recommendation and Comments for Manuscript Number PONE-D-21-13902R1**

**Introduction:**

Para 1 – I recommend opening with a more general statement and simplifying the rest of the paragraph. My suggested revision is below, which you can take or leave as you see fit. I’m not convinced you need the sentence beginning with “Newly published data.”

Becoming a world-class athlete requires systematic, quality training over time (1). Newly published data on elite female and male athletes from different sports indicate that athletes with an average of 10.5 training years have five training sessions and 16 hours of training per week with ~2.5 hours per training session and approximately 18 competitions a year (1). The quality of the training is influenced by the training prescription, which should be in line with the desired outcome (i.e., goal/s), and is defined in terms of training volume, intensity, and frequency (2). Research shows that these three components, collectively referred to as training load, influence training adaptation, and can prevent or cause overtraining, illness, and injury (3). Therefore, the optimal training outcomes depend on an adequate balance between training load components and non-training loads (i.e., stressors) and recovery (4, 5). Ongoing monitoring and modification of these elements is crucial in developing an optimal training prescription that can lead to high-level performance and minimize undesired training outcomes (6-9)*.*

Paras 2-3 – I suggest a bit of simplification and reorganization to clarify your reasoning. Regarding Line 64, (first sentence below), I think “determining” would be a better fit than “deciding” here, as “deciding” implies a choice between a set number of options. Although PLOS ONE asks for “Vancouver” referencing and does not give much direction in terms of formatting and writing style, I think it’s wise to go with the APA standard of restricting the use of “e.g.,” to within parentheses and spelling it out (“For example,”) when outside of parentheses. If you go with my suggestions below, please double-check all of the citation numbers.

When determining the type and amount of training necessary at different stages of an athletic career, it is critical to understand the physiological and psychological demands arising from both the sport (i.e., its physiological and biomechanical profile) and the athlete’s developmental stage. For example, puberty can be a challenging time in athletes' careers, with significant hormonally driven physical changes occurring in both males and females, causing the body to respond differently to exercise. A rapid increase in growth has also been associated with an increased risk of bone and growth plate injuries (12). Moreover, puberty can often be psychologically challenging, especially for females (13). Another potentially challenging period is the transition from lower secondary school to upper secondary school, which typically involves increased training load (14-16) combined with school and other life demands (17). As such, both boys and girls can experience tremendous psychological pressure during this phase (17).

Understanding the sport's demands and the different stages in an athlete's development can help determine the optimal magnitude of the training components to target the desired outcome (i.e., goal/s) at different stages in an athlete's career. Practitioners can then monitor how athletes tolerate training load and make the necessary adjustments to optimize performance capacity (7, 19). Furthermore, reference values can be established regarding training volumes in different sports and recommended progression from year to year, making it easier for both coaches and athletes to design optimal training plans. For example, elite athletes complete between 800–1200 training hours per year in typical endurance sports such as cross-country skiing (20-23), rowing (24, 25), triathlon (26), and swimming (27). In more technically demanding sports such as soccer (28), handball (29), and athletics (30, 31), elite athletes complete between 500–700 annual training hours.

Lines 106, 109, 114 – Replace “where” with “with.”

Lines 109, 112 – Replace “have” vs. “having.”

Line 114 – Could reword as “with higher weekly training volume being associated with more training distress.”

**Materials and methods:**

Lines 142-144 – Update formatting (M ± SD)

Lines 178-180 – Suggested revision: “Cutpoints of 5, 10, and 15 hours of training per week were chosen to ensure relatively equal group sizes”

Line 202 – It would be of great help to students and those of us who do primarily qualitative research if you could include a reminder here of how to interpret these effect sizes (i.e., what constitutes small, medium, and large).

Line 206 – “…of the sample are presented…”

**Results:**

Description of perceived psychological and physiological training distress – Did you include the scale for this instrument somewhere? Is it from 1-5? This would be good to include here.

Line 326 – I think it would be stronger to cut the first part of this sentence and begin with “No significant differences…”

Line 340 – Remove the comma after “sports”

**Discussion:**

Lines 519-527 – This paragraph seems out of place and somewhat detracts from an otherwise strong conclusion. I would recommend incorporating this content into the results section or the first paragraph of the discussion section. For example, “No significant differences in weekly training volume were observed for gender, school program, or school level, contradicting hypotheses 1b and 1c. However, in line with hypothesis 1a, results indicated a significant difference in weekly training volume between sport types, with endurance sports having a higher training volume than more technically demanding sports.”
